# Crustal deformation rates in Kashmir valley and adjoining regions from continuous GPS measurements from 2008 to 2019

**DOI:** 10.1038/s41598-020-74776-5

**Published:** 2020-10-21

**Authors:** Sridevi Jade, Ramees R. Mir, Chiranjeevi G. Vivek, T. S. Shrungeshwara, I. A. Parvez, Rakesh Chandra, D. Suri Babu, S. Vishal Gupta, Ankit, Siva Sai Kumar Rajana, V. K. Gaur

**Affiliations:** 1 CSIR-4PI, CSIR Fourth Paradigm Institute (Formerly CSIR-CMMACS), Wind Tunnel Road, Bangalore, India; 2 Department of Earth Sciences, University of Kashmir, Srinagar, India; 3 Institute of Seismological Research, Gandhinagar, India

**Keywords:** Geophysics, Seismology, Tectonics

## Abstract

We present GPS velocities in Kashmir valley and adjoining regions from continuous Global Positioning System (cGPS) network during 2008 to 2019. Results indicate total arc normal shortening rates of ~ 14 mm/year across this transect of Himalaya that is comparable to the rates of ~ 10 to 20 mm/year reported else-where in the 2500 km Himalaya Arc. For the first time in Himalayas, arc-parallel extension rate of ~ 7 mm/year was recorded in the Kashmir valley, pointing to oblique deformation. Inverse modeling of the contemporary deformation rates in Kashmir valley indicate oblique slip of ~ 16 mm/year along the decollement with locking depth of ~ 15 km and width of ~ 145 km. This result is consistent with the recorded micro-seismicity and low velocity layer at a depth of 12 to 16 km beneath the Kashmir valley obtained from collocated broadband seismic network. Geodetic strain rates are consistent with the dislocation model and micro-seismic activity, with high strain accumulation (~ 7e−08 maximum compression) to the north of Kashmir valley and south of Zanskar ranges. Assuming the stored energy was fully released during 1555 earthquake, high geodetic strain rate since then and observed micro-seismicity point to probable future large earthquakes of Mw ~ 7.7 in Kashmir seismic gap.

## Introduction

**Figure 1:**
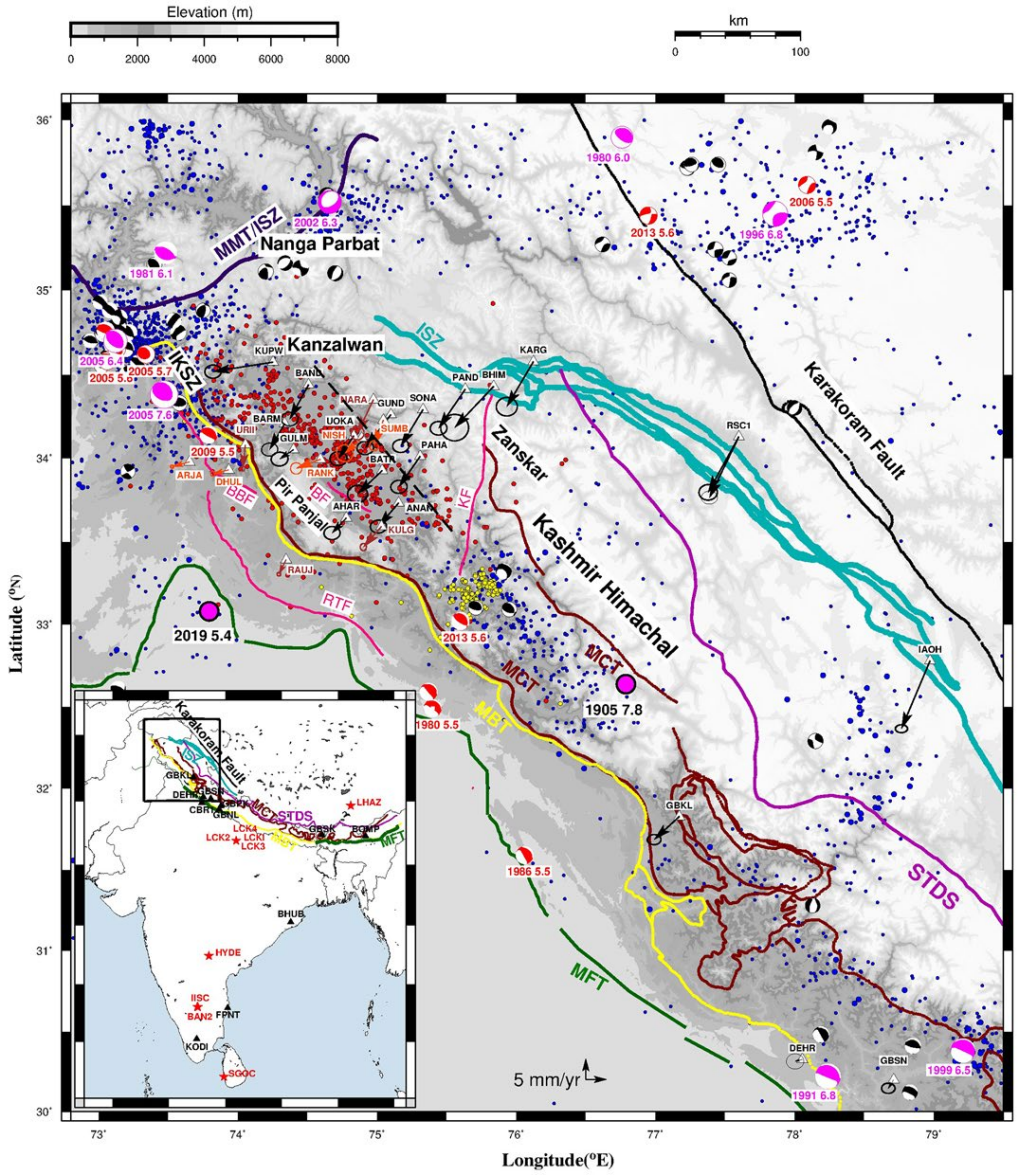
Map of Kashmir valley and adjoining regions (boxed area in the inset map of India) along with major fault/ thrust lines mapped using^41–44^ and seismo-tectonic atlas of India^45^: MFT—Main Frontal Thrust, MBT—Main Boundary Thrust, MCT—Main Central Thrust, STDS—South Tibet Detachment System, ISZ—Indus Suture zone, MMT—Main Mantle Thrust. IKSZ—Indus Kohistan Seismic Zone. Regional fault lines are BBF—Bagh-Balakot fault, RTF—Riasi Thrust fault, BF—Balapora fault and KF—Kishtwar fault. India-fixed velocities of cGPS sites are plotted as arrows: Black (Present study), Red^14^ and Brown^25^. Black dashed line is the apparent northern edge of locked decollement estimated from location of microseismicity using data from collocated broadband seismic network (Supplementary section A1). Solid circles denote seismic events: Blue (M ≥ 3; source: https://www.isc.ac.uk/iscbulletin/search/catalogue/), Red (M ≥ 1 located from collocated broadband network) and Yellow^5^. Focal mechanisms of seismic events of magnitude ≥ 5.0 are plotted from (https://www.globalcmt.org/CMTsearch.html) Global CMT catalogue. Figure was created using GMT (Generic mapping tool) software version 5.2.1 (ftp://ftp.soest.hawaii.edu/gmt/legacy/)^46^.

**Figure 2:**
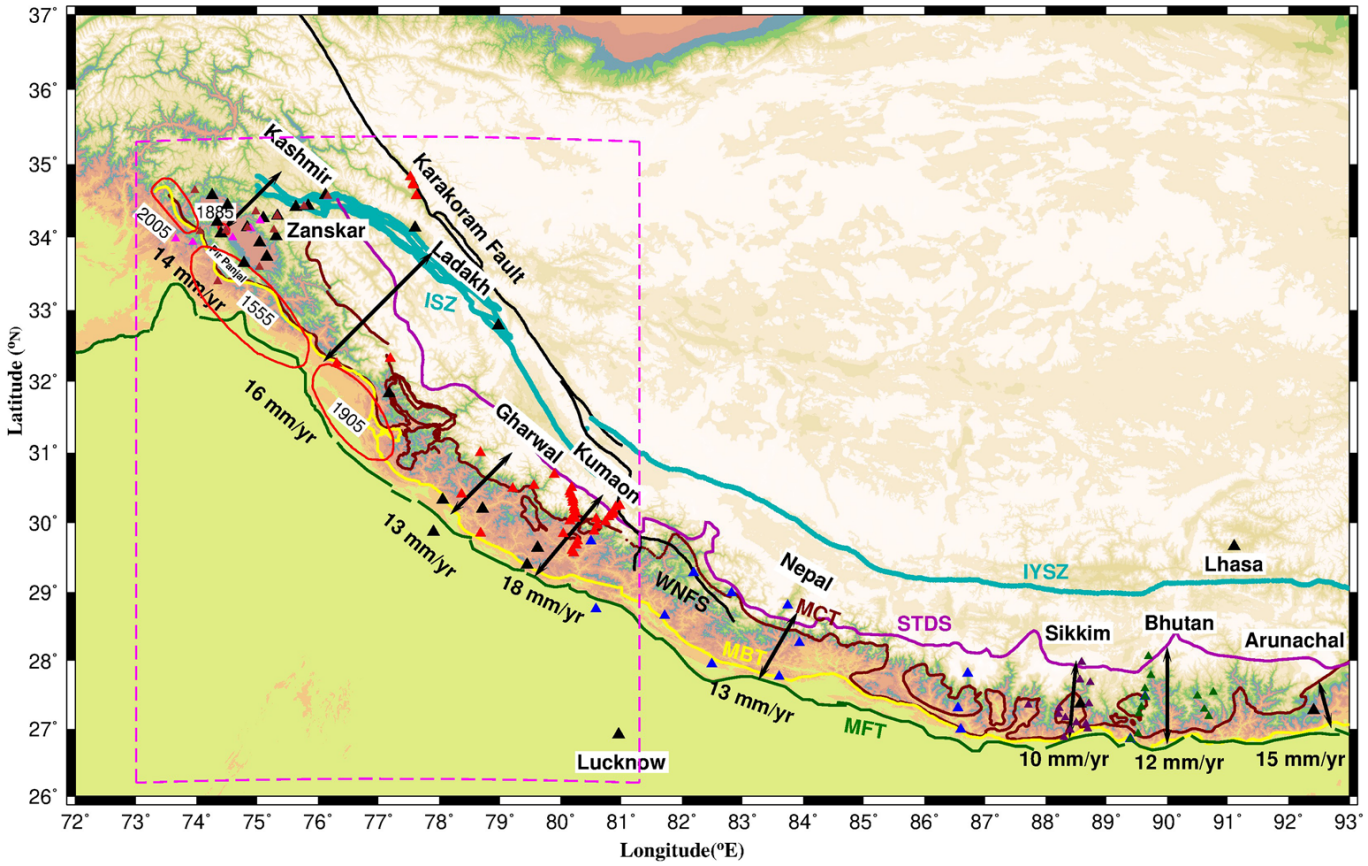
GPS derived surface convergence rates in 2500 km Himalayan arc along with major fault lines mapped using^41–45^: MFT—Main Frontal Thrust, MBT—Main Boundary Thrust, MCT—Main Central Thrust, STDS—South Tibet Detachment System, ISZ—Indus Suture Zone, IYSZ—Indus-Yalu Suture Zone, WFNS—Western Nepal Fault System. Red ellipses represent rupture of 2005 instrumental era event and historical events of 1555 (in Pir Panjal; apparently last major event in the Kashmir seismic gap), 1885 (with epicenter near Pattan, SE of station BARM) and 1905 Kangra earthquake^1–3,7,8,14,42,43,47,48^. GPS site locations are denoted by solid triangles. Black (present study); Magenta^14^; Brown^25^; Red^15^; Blue^19^; dark Brown^20^; Green^21^. Figure was created using GMT (Generic mapping tool) software version 5.2.1 (ftp://ftp.soest.hawaii.edu/gmt/legacy/)^46^.

The 2500 km Himalayan arc from west to east consists of major thrust faults from south to north: Main Frontal Thrust (MFT), Main Boundary Thrust (MBT), Main Central Thrust (MCT), STDS (South Tibet Detachment System) and Indus Suture Zone (ISZ)/Indus Yalu Suture Zone (Figs. 1, 2). The tectonic units bounded by these thrust faults from south to north are Sub Himalaya, Lesser Himalaya, Higher Himalaya, Tethyan Himalaya and Trans Himalaya. These thrust faults sole in to the basal decollement termed as Main Himalayan Thrust (MHT), which marks the upper boundary of under-thrusting Indian plate. Kashmir valley is located to the extreme west of Himalayan arc bounded by Pir Panjal range to the south and Zanskar range to the north (Figs. 1, 2, 4) and it extends about 140 km with a width of ~ 40 km at an altitude of ~ 1600 m. Kashmir Himalaya is tectonically different from the rest of Himalaya with very narrow lesser Himalaya (bounded by MBT and MCT) below the Pir Panjal and wider sub Himalaya. Major known tectonic structures of the study region are Balapora fault (BF), a NW–SE trending thrust structure, in southern region of the basin and Riasi fault (RF) further south of Pir Panjal range, bounding SW side of the valley.

Historical seismicity of Kashmir valley and the adjoining region indicates about ~ 14 damaging earthquakes occurred since year 1123, though the accurate magnitude of these earthquakes could not be determined due to lack of data^1^. Few of these earthquakes with well documented data on intensity of ground shaking suggest magnitude, Mw of 6–8 (Figs. 1, 2, 4). The most recent and first documented historical earthquake in this region occurred on 30 May 1885^2,3^ with an assigned magnitude of Mw 6.2. The 1555 earthquake is the only earthquake between 1123 and 1885 with tentative magnitude of Mw ~ 7.6^4^. Figure 1 shows the earthquakes that occurred in the region from 1964 to 2019. Seismic events are plotted from three sources i.e. M ≥ 3 events with epicentral error of ~ 5 km from 1964 to 2019 from International Seismological Centre (ISC) revised catalogue, local events of M ≥ 1 (for two epochs: June to September 2013; January 2015 to March 2018) with epicentral error of < 5 km from our collocated broadband seismic network and events of M ≥ 1 with epicentral error of ~ 1 km to southeast of the Kashmir valley from^5^. Together these events indicate seismicity cluster to the northwest in IKSZ (Indus Kohistan Seismic Zone) related to post seismic activity of October 2005 Muzaffarabad earthquake, scattered micro-seismicity in Kashmir valley and a cluster related to Mw 5.7 Kishtwar earthquake of 2013 to south east in Kashmir Himachal (or Kishtwar region). Crustal structure beneath the Kashmir valley^6^ delineates the Moho beneath Pir Panjal at depths of ~ 58 ± 2 to 60 ± 2 km and shows crustal thinning of ~ 4 km along the valley axis. Absence of seismicity and duplex nature of topographic height makes it difficult to demarcate the extent of locked MHT in the valley.

The Muzaffarabad earthquake of October 8, 2005 (Mw 7.6) to the northwest of Kashmir valley (Figs. 1, 2, 4) is the first earthquake in Himalaya^7,8^ with well documented surface rupture. This event occurred in IKSZ along the Balakot-Bagh thrust (Figs. 1, 4) and hence termed as an out of sequence event^7,8^ as large Himalayan earthquakes are mostly located along the MHT. Post-seismic deformation of this earthquake^9–11^ was estimated using GPS measurements for a period of 5 years (2005–2010) within 100 km of the rupture and several post seismic relaxation models were suggested. Afterslip rectangular dislocation models^10^ of this earthquake based on Okada dislocation theory^12^ that best fit the GPS displacements cover extreme northwest edge of the Kashmir valley. The ~ 250 km long segment of Kashmir Himalaya (Fig. 1) between rupture zones of the 1905 Kangra earthquake and 2005 Kashmir earthquake has not experienced any major earthquake for past ~ 500 years i.e. since 1555 earthquake and hence termed as Kashmir seismic gap^13^. In 2013, earthquake of Mw 5.7 occurred to the south east of Kashmir valley where micro-seismicity cluster with magnitude of 1–5 were recorded by a recent broadband seismic network^5^. On September 24, 2019, a shallow (depth ~ 10 km), thrust mechanism earthquake of Mw 5.4 occurred in Mirpur, Pakistan bordering south-west of the Pir Panjal range.

**Figure 3:**
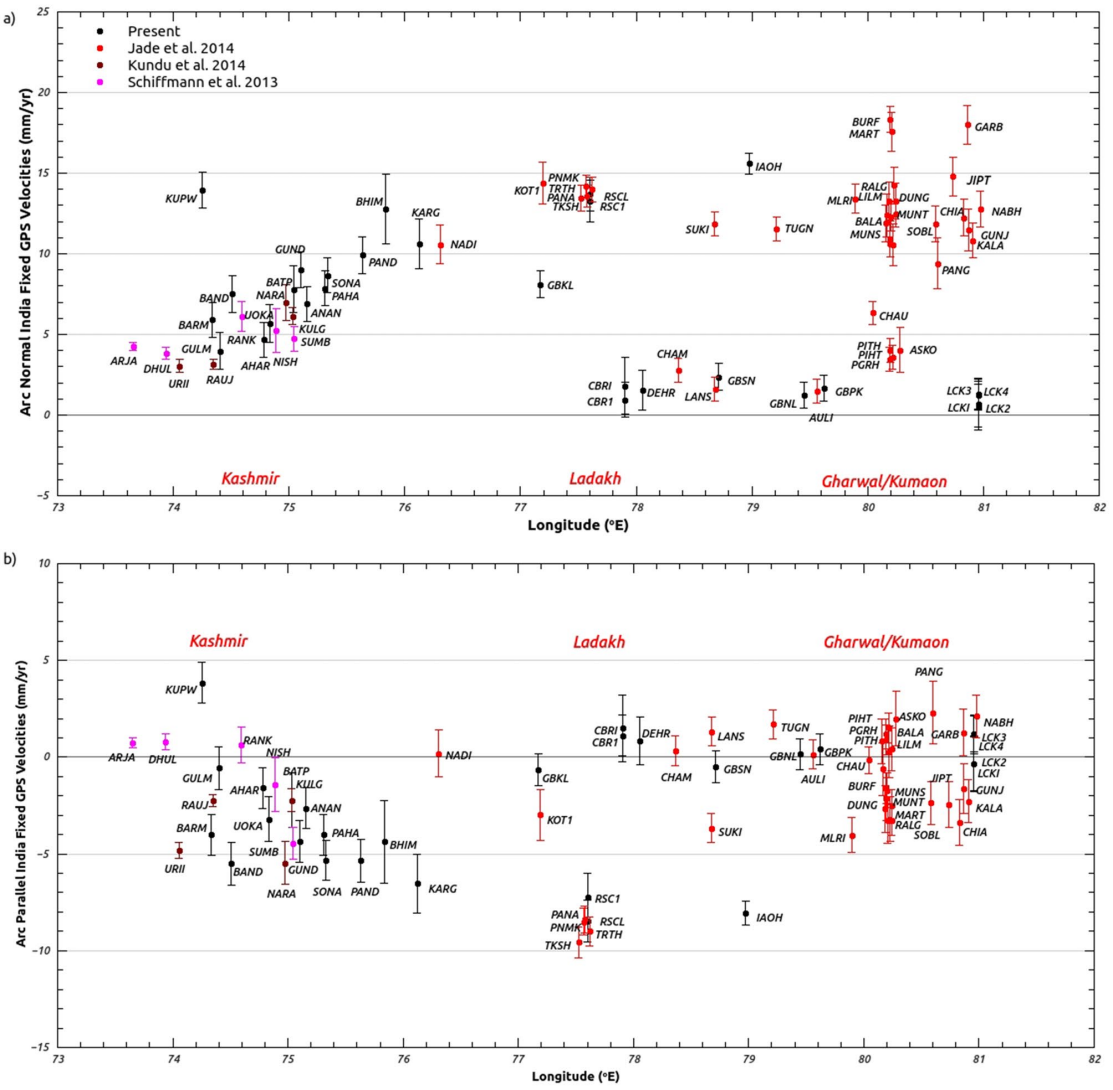
(**a**) Arc-normal rates of GPS sites in northwest Himalaya (boxed region of Fig. 2) with error bars determined by rotating the site velocities on to the direction locally orthogonal to the arc using arc geometry defined by^18^. Figure was created using qtiplot software version 0.9.8.9 (https://sourceforge.net/projects/qtiplot.berlios/files/qtiplot-0.9.8.9.tar.bz2/download)^49^. (**b**) Arc-parallel rates of GPS sites in northwest Himalaya (boxed region of Fig. 2) with error bars determined by rotating the site velocities onto the direction locally parallel to the arc using arc geometry defined by^18^. Figure was created using qtiplot software version 0.9.8.9 (https://sourceforge.net/projects/qtiplot.berlios/files/qtiplot-0.9.8.9.tar.bz2/download)^49^.

Previous GPS studies in Kashmir Himalaya^14^ from 2006 to 2012 indicate ~ 12 mm/year shortening in the 250 km southern most cross section of Kashmir Himalayas. They suggested that earthquake of Mw 6.5–7.6 is long due in this region, depending on the assumption of three failing segments. A decade of GPS measurements in Kashmir-Himachal Himalaya (Figs. 1, 2, 3a) and Ladakh^15^ gave arc normal convergence of ~ 14 mm/year for this transect partitioned to 5 mm/year in lesser Himalaya and 3 mm/year in Higher Himalaya. GPS data from 2008 to 2011^16^ at 7 continuous Global Positioning System (cGPS) and four episodic (eGPS) sites in Kashmir (out of which 3 cGPS and 3 eGPS sites are located in Valley) gave oblique convergence rate of ~ 14 mm/year along the MHT with a locking line located beneath Zanskar range with width ~ 175 km at a depth of 38 km. These studies are inconclusive due to limited data duration from sparse network of GPS sites in Kashmir. We present results from total of 16 cGPS sites data (Table 1) out of which 10 cGPS sites are spatially spread across the length and breadth of Kashmir valley, 4 cGPS located northeast of the valley in Zanskar ranges and 2 cGPS sites further northeast at Leh and Hanle during 2008 to 2019. Our study gives well constrained, precise estimates of crustal deformation and strain rate in Kashmir Himalayan transect with more data and dense network of stations.

**Table 1:** ITRF and India Fixed rates of GPS sites used in the analysis with the location description and the data span.

Site code	Lat (°N)	Long (°E)	Epoch	ITRF14 velocities (mm/year)				India fixed velocities (mm/year)				Description
				N	σN	E	σE	N	σN	E	σE	
**Kashmir/Ladakh Himalayan sites**												
Kupwara KUPW	34.6	74.3	2015–2019	32.26	1.07	18.21	1.09	− 2.23	1.09	− 14.29	1.35	Northwestern part of Kashmir Valley
Baramulla BARM	34.2	74.3	2015–2019	28.33	1.05	29.12	1.07	− 6.17	1.07	− 3.60	1.34	Located on the bank of Jhelum River, also near NW edge of the valley
Gulmarg GULM	34.1	74.4	2015–2019	32.25	1.12	29.52	1.14	− 2.26	1.13	− 3.31	1.39	Located on foothills of Pir Panjal Range SE of Baramulla
Bandipora BAND	34.4	74.5	2015–2019	26.34	1.12	28.20	1.13	− 8.18	1.13	− 4.46	1.38	Located on the NW of Wular Lake
Aharbal AHAR	33.6	74.8	2015–2019	30.94	1.06	29.79	1.07	− 3.61	1.08	− 3.38	1.33	Southwestern part of Kashmir valley, on foothills of Pir Panjal
Srinagar UOKA	34.1	74.8	2015–2019	29.15	1.15	29.29	1.16	− 5.41	1.16	− 3.64	1.41	Located in the centre of Kashmir valley, on western bank of Dal Lake
Batpal BATP	33.9	75.0	2015–2017	28.99	1.41	27.23	1.42	− 5.59	1.42	− 5.88	1.63	NE edge of the valley, NNW of ANAN
Gund GUND	34.3	75.1	2015–2019	26.65	1.09	26.86	1.10	− 7.94	1.10	− 6.09	1.36	Located on bank of Sind river
Anantnag ANAN	33.7	75.2	2015–2019	28.98	1.06	28.44	1.07	− 5.62	1.07	− 4.81	1.33	Southeastern edge of the basin
Pahalgam PAHA	34.0	75.3	2015–2019	27.38	1.06	28.10	1.08	− 7.23	1.07	− 5.05	1.34	Located near Lidder river, Tethys Himalaya
Sonamarg SONA	34.3	75.3	2015–2019	25.92	1.06	27.73	1.08	− 8.70	1.07	− 5.28	1.34	Located on the bank of Sind river, NE of the valley
Pandrass PAND	34.4	75.6	2015–2019	25.35	1.11	26.70	1.12	− 9.30	1.12	− 6.35	1.38	Indus suture zone, Zanskar
Bhimbat BHIM	34.4	75.8	2017–2020	23.77	1.39	24.88	1.40	− 9.79	1.40	− 9.34	1.62	Indus suture zone, Zanskar
Kargil KARG	34.6	76.1	2017–2019	23.97	1.52	26.79	1.53	− 10.73	1.53	− 6.34	1.73	Indus suture zone, Zanskar
Hanle IAOH	32.8	79.0	2008–2019	18.78	0.63	28.18	0.64	− 16.18	0.65	− 6.81	1.00	Mt Saraswathi, Ladakh Himalaya
Leh RSCL	34.1	77.6	2008–2012	20.36	1.07	26.84	1.07	− 14.49	1.08	− 7.03	1.34	Ladakh Himalaya
Leh RSC1	34.1	77.6	2016–2019	21.61	1.27	26.55	1.28	− 13.24	1.28	− 7.32	1.51	Ladakh Himalaya
**Other Himalayan sites**												
Kullu GBKL	31.8	77.2	2008–2014	29.30	0.81	28.88	0.82	− 5.51	0.83	− 5.99	1.12	MCT Zone, on the banks of Beas River
Roorkee CBRI	29.9	77.9	2014–2015	34.68	1.74	33.68	1.75	− 0.20	1.75	− 2.34	1.90	Frontal Himalaya
Roorkee CBR1	29.9	77.9	2015–2019	35.02	1.07	34.56	1.08	0.14	1.08	− 1.46	1.31	Frontal Himalaya
Dehradun DEHR	30.3	78.1	2009–2011	34.44	1.24	34.17	1.24	− 0.45	1.25	− 1.69	1.44	South of MBT in Gharwal Himalaya
Nagoli GBSN	30.2	78.7	2008–2014	32.90	0.82	34.82	0.83	− 2.05	0.84	− 1.30	1.11	Gharwal Himalaya
Almora GBPK	29.6	79.6	2008–2014	34.03	0.80	35.23	0.81	− 0.99	0.82	− 1.41	1.09	Lesser Himalaya of Gharwal
Nainital GBNL	29.4	79.4	2008–2014	34.16	0.80	35.76	0.81	− 0.84	0.82	− 0.94	1.09	Kumaon Himalaya
Panthang GBSK	27.4	88.6	2008–2014	28.01	0.80	39.19	0.81	− 7.19	0.85	− 0.94	1.07	Sikkim Himalaya
Lhasa LHAZ	29.7	91.1	2008–2019	15.24	0.59	46.30	0.59	− 19.85	0.69	6.19	0.94	IGS station located in south-eastern Tibet, China
Bomdilla BOMP	27.3	92.4	2008–2016	19.67	0.73	43.31	0.73	− 15.35	0.82	2.08	1.01	Arunachal Himalaya
**Stable plate interior sites**												
Lucknow LCKI	26.9	81.0	2012–2014	34.34	1.41	38.11	1.42	− 0.76	1.42	− 0.06	1.58	IGS station, Uttar Pradesh
Lucknow LCK2	26.9	81.0	2012–2014	34.53	1.41	38.19	1.42	− 0.57	1.42	0.02	1.58	IGS station, Uttar Pradesh
Lucknow LCK3	26.9	81.0	2015–2019	34.71	0.96	36.48	0.96	− 0.39	0.98	− 1.69	1.18	IGS station, Uttar Pradesh
Lucknow LCK4	26.9	81.0	2015–2019	34.62	0.96	36.49	0.96	− 0.48	0.98	− 1.68	1.18	IGS Station, Uttar Pradesh
Bhubaneswar BHUB	20.3	85.8	2009–2012	35.67	1.17	40.56	1.17	0.42	1.20	− 1.01	1.31	Eastern Ghat Mountains, Orissa
Hyderabad HYDE	17.4	78.6	2008–2019	34.94	0.72	40.83	0.73	− 0.01	0.74	− 0.13	0.92	IGS station in Central India located on deccan Plateau
Bangalore IISC	13.0	77.6	2008–2019	35.34	0.60	42.84	0.60	0.46	0.62	0.79	0.79	IGS station in South India located on Bedrock exposure
Bangalore BAN2	13.0	77.5	2008–2012	34.69	1.01	43.34	1.02	− 0.18	1.02	1.30	1.14	IGS station
Chennai FPNT	12.9	80.2	2016–2019	36.50	1.20	42.20	1.22	1.42	1.21	− 0.23	1.32	Located on Eastern Coastal Plains
Kodaikanal KODI	10.2	77.5	2008–2019	34.86	0.62	43.82	0.63	− 0.01	0.64	1.11	0.80	Southernmost peninsular site
Colombo SGOC	6.9	79.9	2013–2019	35.10	0.84	44.37	0.86	0.04	0.86	0.80	0.98	IGS station, Narahenpita, Colombo, Srilanka
**IGS sites**												
Portblair PBR2	11.6	92.7	2012–2016	19.04	1.04	7.95	1.05	− 15.98	1.11	− 36.31	1.16	Andaman and Nicobar Islands
Karratha KARR	− 21.0	117.1	2008–2019	59.30	0.59	39.05	0.59	29.12	1.05	4.11	0.82	Australian tectonic plate
Wuhan WUHN	30.5	114.4	2008–2016	− 10.59	0.62	33.54	0.62	− 41.59	1.03	− 13.14	0.92	China in Eurasian tectonic plate
Bakosurtanal BAKO	− 6.5	106.8	2008–2019	− 5.96	0.56	24.83	0.57	− 38.91	0.88	− 17.82	0.75	Located in Indonesia
Nanyang NTUS	1.3	103.7	2008–2019	− 5.97	0.96	30.57	0.97	− 39.57	1.14	− 14.01	1.07	Located in Singapore
Kunming KUNM	25.0	102.8	2008–2013	− 16.47	1.50	34.14	1.50	− 50.21	1.61	− 10.34	1.63	China in Eurasian tectonic plate
Cocos COCO	− 12.2	96.8	2008–2019	51.02	0.69	44.23	0.69	16.39	0.83	2.27	0.88	Coco island, western Australian tectonic plate
Selezaschita SELE	43.2	77.0	2008–2013	4.44	0.88	29.17	0.89	− 30.34	0.89	0.53	1.29	Kazakhstan in Tean Shan tectonic plate
Poligan POL2	42.7	74.7	2008–2019	4.29	0.66	28.06	0.68	− 30.24	0.68	0.08	1.15	Kyrghyzstan in Eurasian tectonic plate
Diego Garcia DGAR	− 7.3	72.4	2008–2019	33.96	0.76	47.45	0.79	− 0.31	0.79	2.42	0.94	Diego Garcial Island
Kitab KIT3	39.1	66.9	2008–2018	4.22	0.59	28.55	0.59	− 29.05	0.66	1.36	1.04	Uzbekistan in Eurasian tectonic plate
Seychelles SEY1	− 4.7	55.5	2008–2016	12.73	0.67	24.42	0.66	− 17.65	0.85	− 21.22	0.82	Mahe Island, East African tectonic plate
Bahrain BHR1	26.2	50.6	2008–2009	31.58	1.56	26.66	1.56	2.85	1.68	− 4.18	1.67	Located in Arabian tectonic plate
Bahrain BHR2	26.2	50.6	2008–2009	31.64	1.55	26.67	1.55	2.91	1.67	− 4.17	1.66	Located in Arabian tectonic plate

## Results and discussions

### GPS displacements

**Figure 4:**
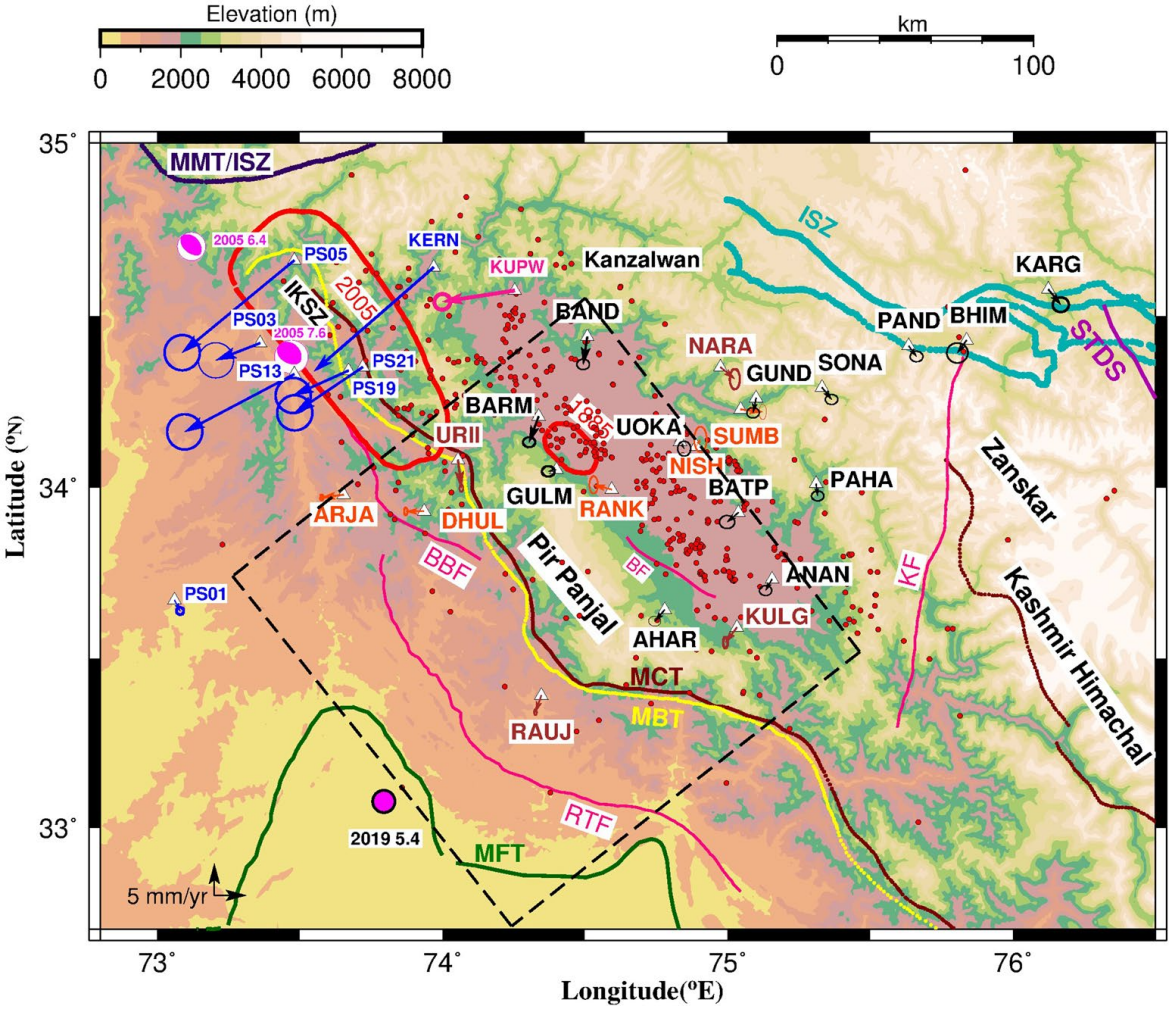
Topographic Map of Kashmir valley and adjoining regions along with surface projection of dislocation plane (dashed box) with residual velocities of the cGPS sites: Black (present study), Red^14^ and Brown^25^. Solid red circles denote seismic events of M ≥ 1 from collocated broadband network. India fixed velocity (bold Pink) is plotted at KUPW GPS site to the extreme northwest of Kashmir valley. Blue velocity vectors at Pakistan GPS sites PS01, PS03, PS05, PS13, PS19, PS21 from^10^ denote post seismic displacements of October 2005 Muzaffarabad earthquake during March 2007 to August 2009 and Indian GPS site KERN^11^ soon after the earthquake. Published post seismic displacements were transformed to ITRF14 reference frame and converted to India fixed reference frame using the angular velocity of Indian plate given by^17^. Fault lines and ruptures are same as Fig. 1, 2. Figure was created using GMT (Generic mapping tool) software version 5.2.1 (ftp://ftp.soest.hawaii.edu/gmt/legacy/)^46^.

Velocities of all the cGPS sites of the present study are estimated using the methodology detailed in Data and Methods section. ITRF14 and Indian plate frame of reference^17^ velocities and the associated uncertainties are given in Table 1. India fixed velocities of cGPS sites in Kashmir valley and adjoining regions are plotted in Fig. 1. Arc normal and parallel deformation rates for sites located in Kashmir valley and the adjoining regions are computed and plotted in Fig. 3a,b. These rates are determined by rotating the site velocities, perpendicular and parallel to the local arc geometry as defined by^18^. In addition, we have plotted in Fig. 3a,b arc normal and arc parallel rates from the published^14,16^ velocities of four GPS sites (ARJA, DHUL, URII, RAUJ) located to south of valley and five sites (RANK, KULG, NISH, SUMB, NARA) located in the valley (Figs. 1, 2, 4). For the rest of northwest Himalaya, we plotted arc-normal and arc-parallel rates from the published GPS velocities of continuous and campaign sites^15,19^. All the published GPS velocities are transformed to ITRF14 reference frame and converted to India fixed reference frame using the angular velocity of Indian plate given by^17^. Arc normal rates indicate total convergence of about 14 mm/year partitioned as 5 mm/year in the Kashmir valley, 5 mm/year south of the valley and 4 mm/year in the Zanskar range north of valley. GPS derived surface convergence rates along the arc normal transects of rest of Himalaya (Fig. 2) from west to east are ~ 16 mm/year in Ladakh, ~ 13 mm/year in Gharwal, ~ 18 mm/year in Kumaon, ~ 13 mm/year in Nepal Himalaya, ~ 10 mm/year in Sikkim, ~ 12 mm/year in Bhutan and ~ 15 mm/year in Arunachal Himalaya^15,19–22^. Kashmir valley recorded arc-parallel extension rate of about 7 mm/year from our cGPS sites (Fig. 3b) spatially spread across the valley indicating that the present day active regional deformation in Kashmir valley is oblique in nature. About 9 mm/year arc parallel rate in Ladakh Himalaya is a result of east–west extension of Tibetan plateau. Rest of the Himalaya records low arc parallel extension rate of ~ 3 mm/year, which is due to curvature of locked decollement^15,19,23,24^.

Kupwara site (KUPW) located to the extreme northwest of Kashmir Valley recorded significantly high westward velocity of 14 mm/year i.e. ~ 8 mm/year relative to rest of the valley sites (Table 1, Fig. 4). Post seismic after slip dislocation models of 2005 Muzaffarabad earthquake in IKSZ zone^7^ suggest post seismic displacements in this region. Afterslip of this earthquake released 56 ± 19% of the seismic moment of main event. GPS measurements^10,11,16,25^ soon after the earthquake at KERN site, located to the west of KUPW (Fig. 4) recorded post seismic displacement of 2005 earthquake. Coordinate time series of KUPW cGPS site in India fixed reference frame from 2015 to 2019 (~ 4.5 years) is given in Figure S5. Though these measurements were made almost a decade after the 2005 Muzaffarabad earthquake, the orientation of India fixed velocity of KUPW site (located ~ 75 km from the epicenter of 2005 event) is similar to the azimuth of post seismic displacement (Fig. 4) recorded at six GPS sites in Pakistan during 2007 to 2009^10^. Further the temporal distribution of seismicity within 100 km radius of the rupture of October 2005 earthquake (Figure S6) for a period of 5000 days (~ 13.7 years) after the earthquake indicated active post seismic deformation in ISKZ, coinciding with period of GPS measurements at Kupwara (2015–2019). This indicates that ~ 8 mm/year westward motion of Kupwara relative to Kashmir valley sites has a component of post-seismic displacement associated with October 2005 Muzaffarabad earthquake.

There is no consensus regarding the geological structure beneath Kashmir valley from previous studies in this region. Deep Seismic sounding recordings in Kashmir Himalaya^26,27^ indicate a complex Moho structure beneath the NW edge of the basin. For example^28^, reported Moho depth of 45 km beneath the Sopore (~ 37 km SSE of Kupwara; ~ 15 km NNE of BARM) and few km to the north Moho dips to a depth of 54 km near BAND cGPS site. Further^27^, reports that Moho dips to ~ 65 km beneath the Kanzalwan (North of Bandipora town), and further up-warping to the depth of ~ 60 km beneath the Nanga Parbat. The Moho offsets beneath the Sopore and Kanzalwan are ascribed to 2 deep rooted faults beneath the region, apparently oriented in NW–SE direction. The processed broadband data from our network (Supplementary section A1) could not find any evidence of the Moho dip between Sopore and Bandipora. However, increase in Moho depth from ~ 54 ± 2 km beneath the Sopore to ~ 64 ± 2 km north of Kanzalwan is clearly observed, indicating ~ 11° dip of the Moho along with a similar dip of the decollement underneath (Supplementary section A2). This step in decollement and Moho may represent one of the deep rooted faults described by^27^ which may have contributed to the ~ 8 mm/year westward velocity of KUPW in addition to the post seismic component of 2005 Muzaffarabad earthquake which can be only be confirmed by establishing additional cGPS sites in this region.

Three cGPS sites (PAND, BHIM, and KARG) located in the Indus Suture Zone (ISZ) (Fig. 1, Table 1) record relative motion of ~ 3 mm/year indicating active regional deformation in this region. Collocated broadband seismic data (Supplementary section A3) indicates upwarping of the Moho from the depth of 66–68 km beneath Pandrass (PAND) to 60 km beneath Kargil (KARG). This undulation of about 6 km of the Moho in arc normal direction requires further corroboration in terms of the subsurface structures in this region. Present day regional deformation rate of ~ 3 mm/year recorded in ISZ may be due to the presence of active unmapped subsurface structures in this region. Confirmation or rejection of this hypothesis would require further cGPS and broadband seismic data from the region. Further, a similar Moho depth of ~ 68 ± 2 km is obtained beneath surface expression of ISZ at different points from Kashmir broadband data. A similar depth of the Moho (~ 70 km) was also observed beneath the ISZ in Nepal Himalaya^29^. However, seismic data points towards more complex crustal structure beneath the Zanskar as compared to the Kashmir valley (Supplementary section A3).

Surface projection of Balapora fault (Fig. 1) in Kashmir valley coincides with southwest end of Kashmir valley seismic line defined by^26,27,30^. Balapora fault is northeast dipping reverse fault with 40 km surface length^31^ where as it is also been interpreted as surface expression of SW dipping back thrust^32^. Aharbal cGPS site is located to the south of Balapora fault (~ 12 km) and Batpal site is located to the north (~ 27 km). Baseline between these two sites indicates convergence rate of about 3.5 ± 1 mm/year. Given that, cGPS sites (UOKA, RANK, GULM, BARM) located in Kashmir valley to west of Balapora fault do not record significant convergence across the valley suggesting that the fault may be active and is accommodating about 3.5 mm/year convergence in the Kashmir valley. Earlier study^14^ reported that the entire long-term convergence in this region is absorbed by Balapora, Riasi and MFT faults.

Broadband seismic network in the valley recorded Mw 5.4, 24 September 2019 earthquake in Mirpur, Pakistan to the south of Jammu and Kashmir. For example, maximum absolute peak velocity of 0.688 mm/s was recorded at the nearest station AHAR on N–S component and slightly less on both E–W (0.559 mm/s) and vertical (0.517 mm/s) components for this event. This is also evident from earthquake intensity map which reported higher intensity in NW–SE direction of the event, more towards SE direction (https://earthquake.usgs.gov/earthquakes/eventpage/us60005mqp/map). However, both 30 s and 1 s interval cGPS data of Kashmir valley did not record any co-seismic offset of Mw 5.4 2019 earthquake with epicenter at about 100 km southwest of the valley.

### Dislocation models

Inverse modeling using Okada dislocation theory and weighted least square inversion^12^ of India fixed velocities of Kashmir valley and the adjoining regions (Table 1) provide buried dislocation parameters and the associated slip. Assuming that the surface deformation is caused due to slip along the single planar dislocation (MHT) of finite length and width in elastic half space, the equation for slip, say *m* following^12^ is given as *m* = (G^T^·W_e_·G)^−1^ G^T^·W_e_·d, Where G is a function of dislocation parameters, W_e_ is weight matrix, d are GPS velocities. The slip *m* consists of two orthogonal components, dip-slip and strike-slip. The weight matrix is taken as the inverse of covariance matrix of the observed GPS velocities. Input to the inverse program are the range of a-priori dislocation parameters (starting point, dip, strike, depth, length, width), and GPS velocities. The output is the slip estimated using inversion technique for all possible combination of dislocation parameters. Best fit is the solution for which the residual between the observed and modelled displacements is minimum (Fig. 4). Complete detail of the dislocation modeling theory is given in^15^.

We used the GPS velocities from our study along with the published velocities^14,25^ of 9 cGPS sites in this region (Fig. 4). The best-fit dislocation model for Kashmir valley gives a dip-slip of ~ 15.5 mm/year and strike-slip of ~ 4 mm/year along the single buried dislocation with a depth of ~ 15 km, length and width of ~ 145 km. Dip and strike angle of the buried dislocation is 7° and 142° respectively (Fig. 4). Micro-seismic events recorded by collocated broadband network in Kashmir valley and adjoining regions are plotted in Figs. 1 and 4 along with seismic events from ISC catalogue (Fig. 1) and one year local events from^5^. Micro-seismicity in Kashmir valley from collocated broadband data (Supplementary Section A1) points towards probable location of the northern edge of locked decollement plotted by dashed black line in the Fig. 1. In addition, results from Kashmir broadband network (Supplementary Section A1) found a low velocity layer at a depth of 12–16 km representing the locked decollement^6^. Our dislocation model is consistent with the northern edge of locked decollement, as highlighted by location of micro-seismicity, and the depth of decollement reported from Kashmir broadband network data.

Previous studies with limited spatial spread and duration of GPS data^16,25^ and using^33^ elastic dislocation theory, reported oblique slip of ~ 13.6 mm/year with dip slip component of 11.8 mm/year and strike slip component of 6.7 mm/year in Kashmir valley along locked decollement of 175 ± 20 km width, (38 km depth) and dip of 12.5°. Boundary element method was used^14^ to identify subsurface geometry and slip distribution that best fits the GPS velocities from the limited GPS data. Their GPS data gave good constraints on the slip rate of 11 mm/year along a single planar dislocation with a dip of 7°. They could not constrain the depth of dislocation and it varies between 15 and 23 km. They also gave an alternative model with double ramp geometry with tapered slip with a width of 35 km terminating beneath the north edge of Kashmir valley at 25 km depth that fits the surface GPS velocities with a slip of 11 mm/year^14^. Our GPS measurements with good spatial spread and long duration data give well constrained depth of dislocation as 15 km and oblique slip of ~ 16 mm/year which is consistent with the presence of low velocity layer of 12–16 km depth interpreted as shear zone associated with locked decollement^6^. Diffused seismicity at shallower depths is observed to the southeastward of the valley at longitude 75.75° E.

### Strain rates

Our surface GPS velocities along with published GPS velocities at 9 additional sites^14,16,25^ were used to determine the crustal strain rates in Kashmir valley and adjoining region. Displacements (U_*n*_) and position (X_*n*_) of *n* GPS sites are used to determine the strain tensor (I) using least square approach expressed as U = AI + *e* in matrix form, where matrix *A* is given by the position of GPS points and *e* is the residual vector^34^. Modified least square method^35^ is used in which adjustment based on the effect of nearest sites is made on the least square covariance matrix using a scale factor. This gives the choice to use different scales of analysis and evaluate the scale-dependent behavior of the observed system and select a scale factor that best fits the observed system. Grid-Strain program^34^ enables to include or exclude a GPS point to estimate the effect of the specific point and is advantageous to compute strain rates using unevenly distributed cluster of GPS points. The inputs to the program are GPS velocities and positions and the output is the strain tensor.

**Figure 5:**
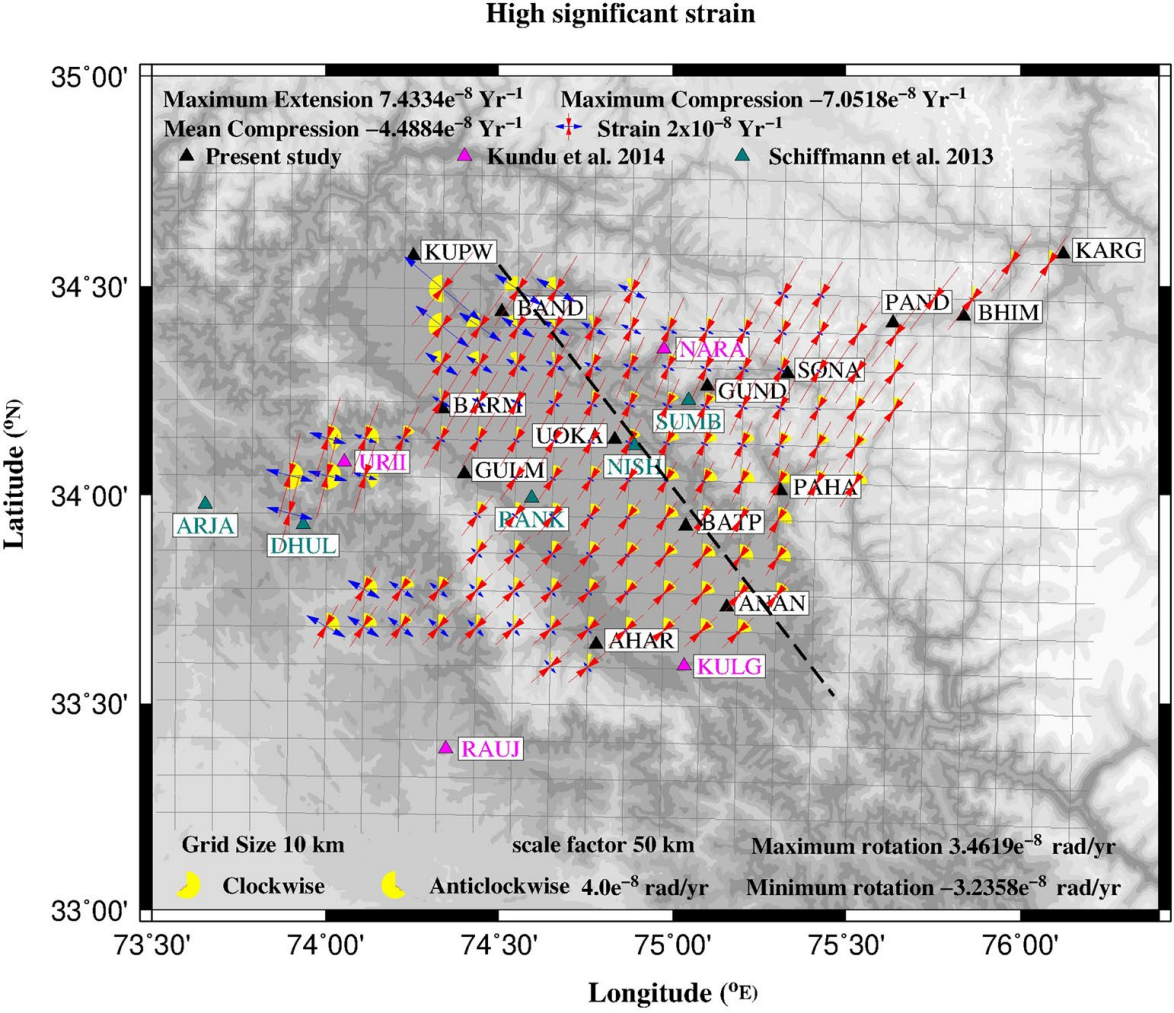
High significant strains at each grid point (resolution: 10 × 10 km). Red: Compression, Blue: Extension, Yellow: Rotation. cGPS site velocities used for determining the strain rates are denoted by solid triangles. Black dashed line is the northern edge of locked decollement estimated from dislocation model. Figure was created using GMT (Generic mapping tool) software version 5.2.1 (ftp://ftp.soest.hawaii.edu/gmt/legacy/)^46^.

We used two dimensional grid strain program in which computations are performed at each node of *XY* grid (Fig. 5) and the output given as strain field tensors consisting of eigen vectors, eigen values and the normalized shear. Strain at a point is considered highly significant if the distance between the reference point and at least one GPS point in that sector is less than the scale factor chosen for the observed system. High significant strain for Kashmir valley along with associated rigid rotations are plotted in Fig. 5, which indicates high compression (red) with a mean rate of 4.5e−08 year^−1^ approximately normal to the arc in Kashmir valley and adjoining regions. Maximum compression of 7e−08 year^−1^ occurs along the northern edge of decollement. High extension (blue) rate of 7.4e−08 year^−1^ is observed to the northwest of the valley which is due to the anomalous westward motion recorded at KUPW GPS site. In Zanskar range and ISZ the strain rates are higher compared to the valley. Demarcation of strain rates to the south of Zanskar and north of valley indicates high strain accumulation in this region that coincides with the northern boundary of locked decollement. Our results are broadly consistent with that of^16^ indicating high strain concentration in southern Zanskar Himalayan region with the limited data.

## Conclusions

cGPS measurements in Kashmir Himalaya and adjoining region give well constrained oblique surface deformation rate of ~ 15.6 mm/year in this transect of Himalaya. This region records total arc normal GPS convergence rate of 14 mm/year and arc-parallel extension rate of 7 mm/year. Elsewhere in the Himalaya, from Ladakh in the west to Arunachal in the east record ~ 10 to 20 mm/year arc-normal convergence with lowest rate of ~ 10 to 12 mm/year in Sikkim and Bhutan Himalayas. Additional cGPS measurements are required (1) to precisely quantify the contribution of post seismic component of 2005 Muzaffarabad earthquake to the high westward velocity recorded to the extreme northwest of Kashmir valley, (2) to estimate the slip rate along Balapora fault and (3) to better constrain the crustal deformation in ISZ.

Inverse modeling of GPS velocities in Kashmir valley and adjoining regions give oblique slip of ~ 16 mm/year along the 145 km wide decollement at a depth of 15 km. Our study using good spatial spread and long duration cGPS data gave reliable constraints on the depth of decollement which is consistent with the recorded seismic activity and low velocity structure^6^ mapped around same depth (12–16 km). Strain rates estimated using the GPS velocities indicate high strain accumulation (7e−08, compression) to the north of the valley and south of Zanskar consistent with the northern edge of the locked decollement mapped using the seismic activity and inverse models. Strain rates (extension) to the SE of Kupwara are due to the anomalous westward GPS velocity of KUPW cGPS site which needs further investigation.

Historical earthquake record suggests about 14 damaging earthquakes occurred during 1123 to 1885, with only one earthquake of Mw ~ 7.6 in 1555 with poorly estimated rupture parameters. Kashmir seismic gap of ~ 250 km length between the rupture zones of 2005 Muzaffarabad and 1905 Kangra earthquakes did not record any major seismic slip during the intervening ~ 465 years. Considering that all the accumulated strain is fully released during the 1555 earthquake and fresh strain accumulation started since then, high strain rates recorded in this region give a scalar geodetic moment accumulation rate^36^ of ~ 8.91e24 dyne-cm/year (~ 4.14e27 dyne-cm for 465 years) which is capable of generating Mw 7.7 earthquake. Accumulated slip deficit since 1555 event is ~ 7.4 m (16 mm/year for 465 years) and assuming rupture of the probable earthquake to be similar to 2005 Muzaffarabad earthquake, seismic moment^37^ is ~ 5.3e27 dyne-cm suggesting Mw 7.8 earthquake is long due in this region. If the seismic moment released due to the after slip of the 1555 event is assumed to be similar to the 2005 earthquake^10^, the reduction in the Mw for a single event would be ~ 0.14. Micro-seismicity recorded in Kashmir valley further corroborate that a high magnitude earthquake is long due in this region. Our hypothesis is broadly consistent with the conclusions drawn by^6,14^ that large magnitude earthquake is anticipated in this region, may be with low probability of occurrence.

## Methodology

### GPS data and analysis

Complete details of cGPS sites and data span used for the study is given in Table 1. In addition to 16 cGPS sites in Kashmir we have used cGPS data of 9 sites in the rest of Himalaya and 10 plate interior sites (Fig. 1). All the cGPS sites are located on hard rock exposures (except the Kashmir University (UOKA) cGPS site) with 3–10 years of data. cGPS data spanning 12 years (2008–2019) is analysed using GAMIT/GLOBK software^38^ along with 22 IGS sites (Table 1) after performing quality check on the data using TEQC software^39^. Loosely constrained daily solutions were obtained using sampling interval of 30 s and elevation cut off angle of 15° after eliminating data with short duration (< 20 h), multipath and several cycle slips. Errors related to satellite clock, receiver clock, phase ambiguities, atmosphere, phase center and multipath are minimized in the daily solutions as detailed in^19^. Final daily solutions are combined using GLORG to determine the positions and velocities of the cGPS sites in ITRF2014 reference frame^40^ by stabilizing the IGS sites to their ITRF2014 positions and velocities. ITRF2014 velocities and the associated uncertainties are given in Table 1 for all the cGPS sites used in the present study. India fixed velocities (Table 1) and associated uncertainties of the cGPS sites are determined using the angular velocity of India-ITRF14 as given by^17^.

## Supplementary information

Supplementary file1
