# Supplementary material for: Crustal deformation rates in Kashmir valley and adjoining regions from continuous GPS measurements from 2008 to 2019
Source: Sci Rep. 2020 Oct 21;10:17927. doi: 10.1038/s41598-020-74776-5 (PMC7577991; doi:10.1038/s41598-020-74776-5)
Supplement: Supplementary file 1 — Supplementary file1 [file 41598_2020_74776_MOESM1_ESM.docx]

Crustal deformation rates in Kashmir valley and adjoining regions from continuous GPS measurements from 2008 to 2019

**Sridevi Jade^1,*^, Ramees R Mir^1^, Chiranjeevi G Vivek^1^, T. S. Shrungeshwara^1^, I.A.Parvez^1^, Rakesh Chandra^2^, Suri Babu D^3^, S. Vishal Gupta^1^, Ankit^1^, Siva Sai Kumar Rajana^1^ and V. K. Gaur^1^**

1 CSIR-4PI, CSIR Fourth Paradigm Institute (formerly CSIR-CMMACS), Wind Tunnel Road, Bangalore – 560 037, India

2 Department of Earth Sciences, University of Kashmir, Srinagar, India

3 Institute of Seismological Research, Gandhinagar, India.

*Corresponding author. Tel.: (+91) 80-25051929 Fax: (+91) 80-25220392

E-mail addresses: sridevi@csir4pi.in (Sridevi Jade); ramizmir752@gmail.com (Ramees R Mir); vivek@csir4pi.in (Chiranjeevi G Vivek); shrungeshsringeri@gmail.com (T. S. Shrungeshwara); parvez@csir4pi.in (Imtiyaz Ahmed Parvez); [rakeshchandra69@gmail.com](mailto:rakeshchandra69@gmail.com) (Rakesh Chandra); suribabu.9396@gmail.com (Suri Babu D); [svishal311.bhu@gmail.com](mailto:svishal311.bhu@gmail.com) ( S. Vishal Gupta); [ankit.pathania72@gmail.com](mailto:ankit.pathania72@gmail.com) (Ankit); sivasai7552081@gmail.com ( Siva Sai Kumar Rajana), [gaur@csir4pi.in](mailto:gaur@csir4pi.in) (V. K. Gaur)

**Supplementary Information**

This supplementary section contains:

An introduction to seismic data methodology followed to generate receiver function, to obtain shear-wave velocity structure underneath selected broadband seismic stations and location of the MHT.

Possible mechanism to explain ~8 mm/yr westward motion of Kupwara cGPS site located to extreme northwest of Kashmir valley and ~3 mm/yr relative regional deformation recorded by 3 cGPS sites located in Indus Suture Zone (ISZ).

GPS coordinate time series of Kupwara cGPS site (Figure S5)

Seismicity plot for a 14 year period following October 2005 Muzaffarabad earthquake (Figure S6)

**A1: Seismic Data and Methods**

Teleseismic earthquakes, having epicentral range of 30-100°, were recorded at 14 seismic stations in Kashmir basin and adjoining regions of Zanskar from 2013-2019 (Figure S1). These waveforms were deconvolved (radial/vertical), in time domain, and passed through a low pass Gaussian filter of width 2.5Hz, providing a vertical resolution of ~1.6 km. The resulting waveforms are sensitive to velocity contrasts beneath a receiver, hence called Receiver Functions (RFs). Quality control and RF generation is discussed in detail in Mir et al. (2017). RFs thus generated were jointly inverted with fundamental mode Rayleigh-wave group velocity dispersion measurements obtained from ~2500 source-receiver paths, from regional earthquakes (Mir, 2020). Constrains on the Moho depths were obtained by employing a bi-fold forward modeling method (Mir et al. (2017)). The position where the individual incident, near vertical, teleseismic waveforms ‘pierce’ the Moho – Moho piercing points, beneath each station are plotted as red crosses (Figure S1), by assuming a Moho depth of 54 km beneath the region. This assumption was provided both by deep seismic sounding data of Kaila et al. (1984) as well as slant-stack method employed by Mir et al. (2017) by assuming a fixed P-wave velocity of 6.27 km/s.

Receiver Functions are constituted of direct waveforms, traversing velocity contrasts like the Moho, MHT etc., and their time-delayed reverberations. The negative phase at ~1-2 sec on Receiver Functions from the Kashmir basin (lower part of Figure S4) is interpreted as signal from the MHT, which corresponds to a negative gradient in shear-wave velocity with respect to depth at ~12-16 km. Various authors suggested different reasons for this negative gradient of velocity ranging from presence of water at this depth released due to sediment cover of Indian plate to trapped fluids in fault gouge etc. Constrains on thickness of the MHT are itself provided by the vertical resolution (~1.6 km) of RFs. Detailed discussion on arguments supporting the identification of the MHT from receiver functions in Kashmir basin is given in Mir et al. (2017).

Bollinger et al. (2004) argued that geodetic measurements in the Himalaya point towards uniform pattern of inter-seismic strain unlike patterns of micro-seismicity. Bollinger et al. (2004) and earlier Cattin and Avouac (2000) both concluded that the location of micro-seismicity is controlled by the inter-seismic stress buildup. The uneven distribution of the former reflects towards heterogeneous buildup of the latter across the Himalaya. The presence of scattered micro-seismic events (Figure S2) within the Kashmir basin bounded on north by the foothills of Zanskar range thus represent a stress buildup apparently since 1555 earthquake, whose aftershocks were felt for atleast for a month. Micro-seismicity from collocated broadband seismic data in Kashmir points towards probable location of the northern edge of locked decollement as shown in Figure S2.

**A2: Step in the Moho and decollement north of Bandipora (south of Kupwara): An apparent location of a subsurface fault**

Forward modeled Moho depths beneath NW edge of the basin (~17 km E of station BARM, ~45 km SE of KUPW) from events recorded at stations BARM and GULM are 54±2 km to 56±2 km (Figure S3). However, RFs at station BDP (~10 km east of cGPS site BAND) between back-azimuth of 330°-70° required Moho depths of 64 to 66±2 km (Figure S3), implying Moho dip of ~11° towards NE in this region. This dip in Moho is absent beneath SE edge or middle of the valley. Further, we processed data from nearby stations along an arc-normal profile crossing NW of the valley (L1-L1’, Figure S1), starting from the MBT. Here, RFs within a swath width of ±30 km from the profile L1, and 2 km wide bins were stacked, with zero overlap, to improve signal to noise ratio. The resulting plot for L1-L1’ is shown in Figure S3 (bottom). Numbers plotted on Y-axis for each RF (at ~14.5 sec) are the number of RF(s) stacked to generate given RF for each bin. Thus, both forward modeling of individual RF and SW joint inversions as well as stacked RFs plotted along an arc-normal profile does confirm the step in decollement as well as the Moho in NW of the valley as marked by dashed lines. Further, detailed subsurface mapping of this structure and its affect, if any, to contribute to ~8 mm/yr westward rate at KUPW cGPS site will only be confirmed by a careful installation of additional broadband and cGPS sites.

**A3: Moho dip beneath Bhimbat, Drass and further upwarp beneath Kargil.**

RFs at PAND site with back-azimuth of ~80-90° pierce Moho beneath the station BHIM. Forward modeling suggests a Moho depth of 66-68±2 km (Figure S4; Mir, 2020) from these RFs, apparently being close to the ISZ. However, Moho depths estimated from station KARG suggest towards Moho upwarp (58-60±2 km) underneath (Figure S4). Stacked RFs along NE-SW cross-section passing near stations BHIM and KARG also reveal this step, and a complex structure underneath. RFs along this profile were also stacked with bin width of 2 km starting from the MBT as described for Kupwara profile. This unique disposition of the Moho needs further corroboration from more seismic and cGPS data.

**
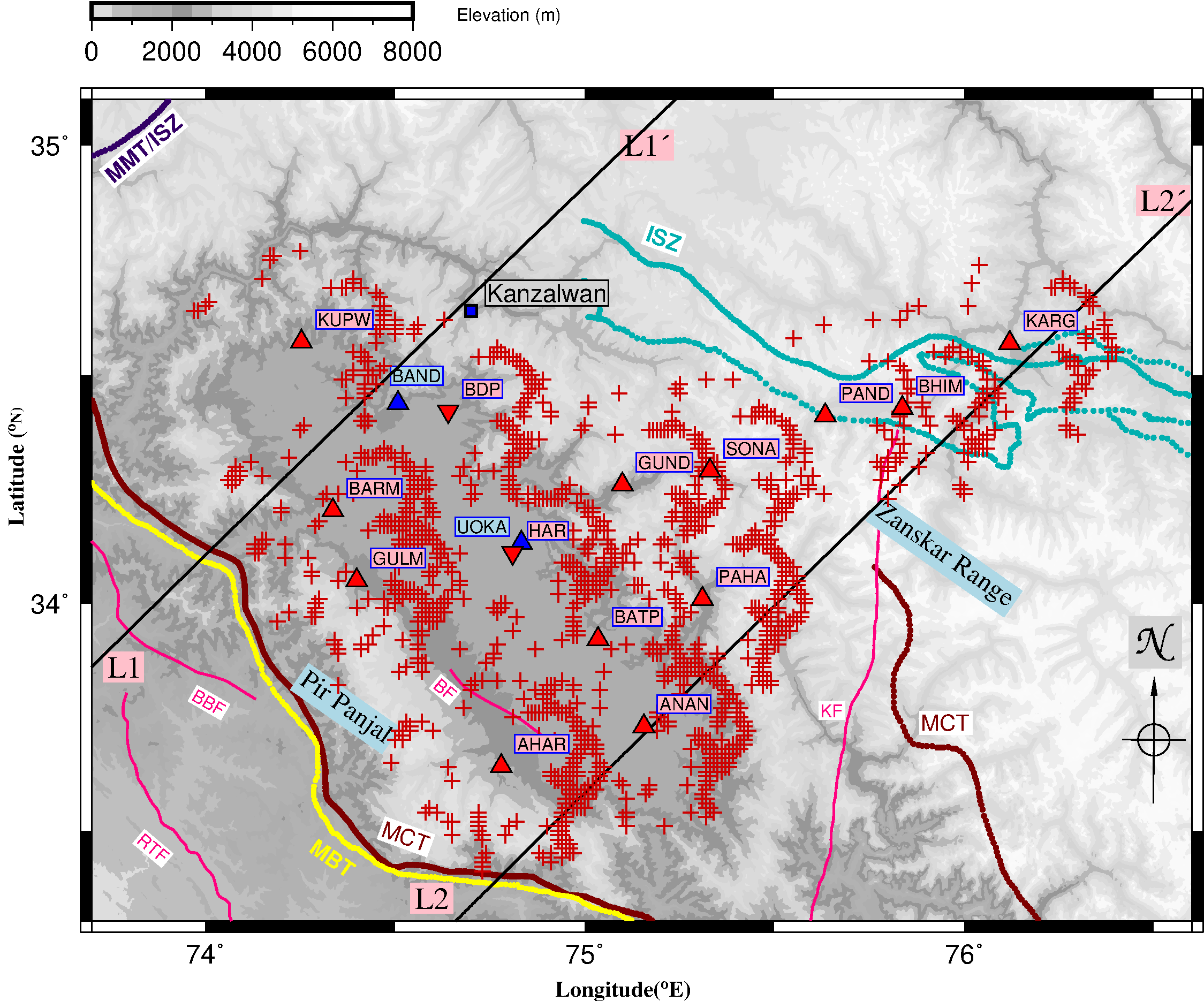
**

**Figure S1:** Shows map of study region. Red triangles represent the location of both seismic and cGPS stations, while as sites HAR and BDP are only equipped with broadband seismic instruments hence shown by inverted red triangles. Two sites which were only equipped with cGPS stations are UOKA and BAND, shown by blue triangles. Blue square represents the location of Kanzalwan. Red crosses are Moho piercing points corresponding to RFs used in this study. L1-L1`and L2-L2` are arc-normal profiles along which stacked RFs are plotted in Figure S3 and S4. MBT - Main Boundary Thrust, MCT - Main Central Thrust, ISZ - Indus Suture zone, MMT - Main Mantle Thrust. Regional fault lines are BBF - Bagh-balakot fault, RTF - Riasi Thrust fault, BF - Balapore fault, KF - Kishtwar fault. Figure was created using GMT (Generic mapping tool) software version 6.0.0 (http://gmt.soest.hawaii.edu/projects/gmt/wiki/Download) (Wessel, P. et al. 2019)

**
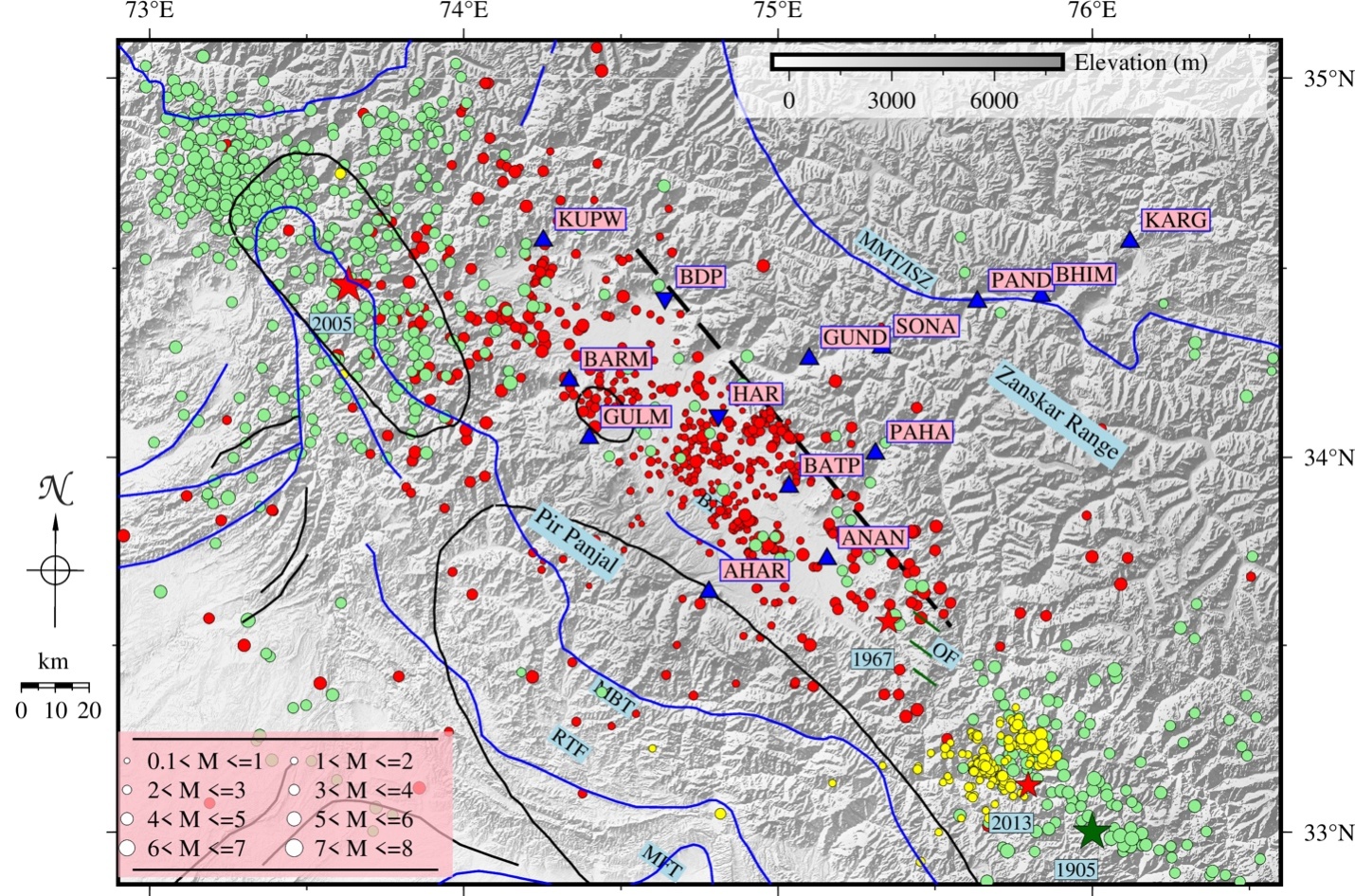
**

**Figure S2:** Map of study region with relocated seismicity from ISC catalog (green circles; M≥3; 1964-2019), local micro-seismicity from our broadband network with epicentral error of <5 km (red circles; M=0.2-5.0; 718 events with 38 events M>3) and 1 year of micro-seismicity (yellow circles; M=1-5; 218 events with 24 events M>3) in Kishtwar region from Paul et al. (2018). All events are scaled according to their magnitude (see legend). Dashed line represents apparent location of the northern limit of the locked decollement encompassing most of the micro-seismicity in the Kashmir basin, representing stress buildup in the region. Local micro-seismic events (red circles) are obtained for two epochs: June to September 2013 and January 2015 to March 2018. Rupture ellipses are same as Figure 2 of main text. Triangles (four letter coded) represent sites where GPS and seismic stations are collocated. Inverted triangles (three letter coded) represent sites which are only equipped with seismometers. Red stars represent location of Mw 7.6 2005 Kashmir event and 2013 Mw 5.7 Kishtwar event. Green star represents epicentral location of Mw 7.8 1905 Kangra event. All blue lines represent thrust faults viz. MFT -Main Frontal Thrust, MBT - Main Boundary Thrust, ISZ/MMT - Indus Suture zone: Main Mantle Thrust, BF - Balapore Fault and RTF- Riasi Thrust fault. Three normal faults SE of the valley are Oldham’s Faults (OF). All unclassified faults are marked by black lines.

Figure was created using GMT (Generic mapping tool) software version 6.0.0 (http://gmt.soest.hawaii.edu/projects/gmt/wiki/Download) (Wessel, P. et al. 2019)


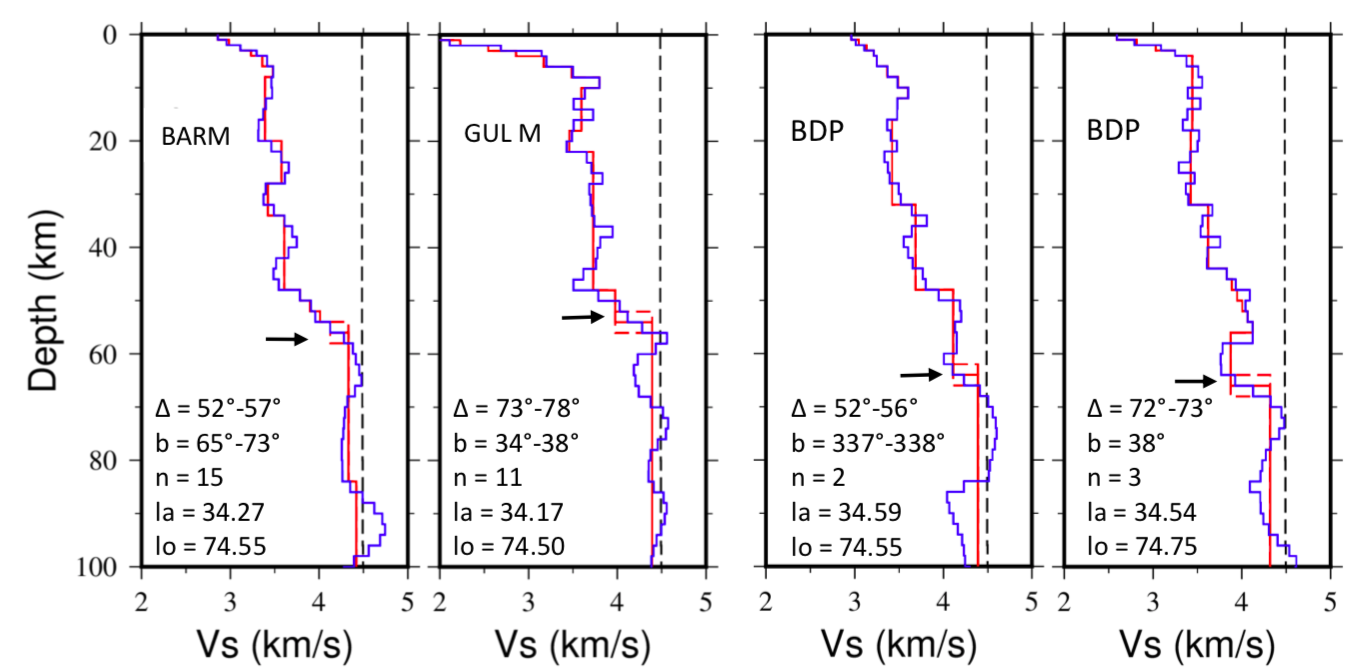

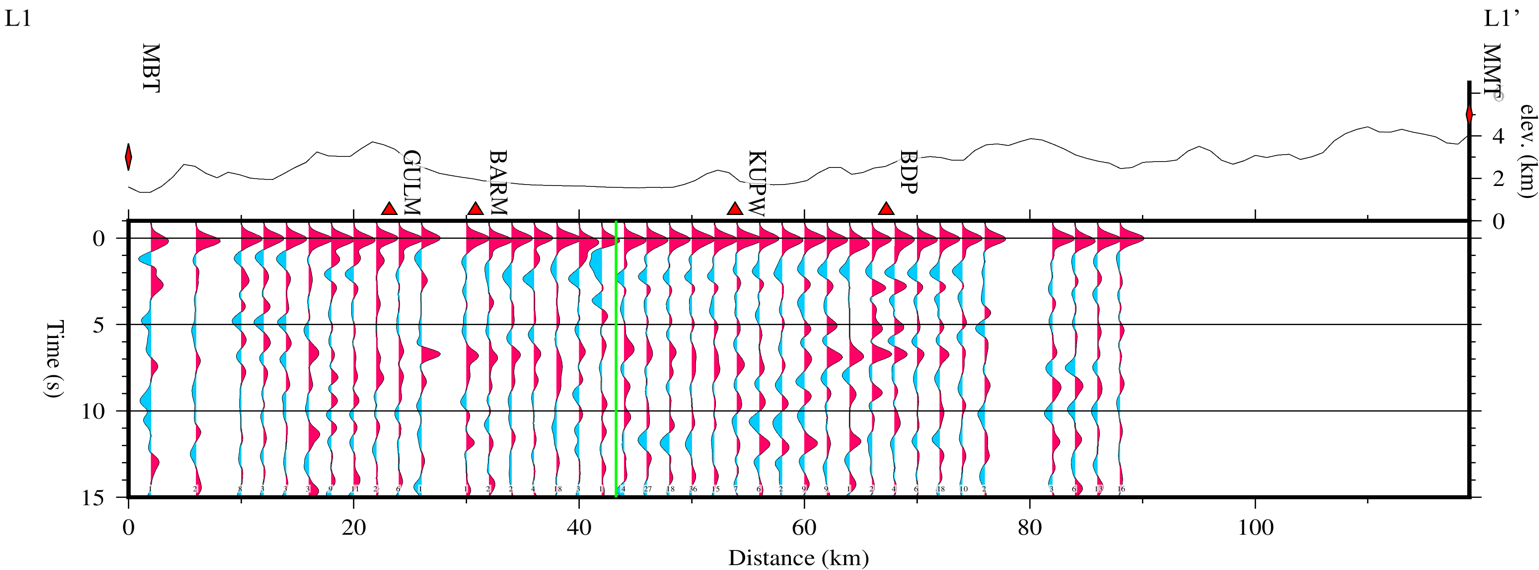


?

?

**Figure S3**: (**TOP)** shows 4 shear-wave velocity models upto depth of 100 km obtained from joint inversion of stacked receiver functions (RF) and fundamental-mode group-velocity Rayleigh-wave dispersion (SWD) (8-60 sec). In each case, dashed black line represents a starting model with fixed Vs of 4.48 km/s, close to upper mantle velocity to have no bias while performing the inversion. Blue line represents the model obtained from joint inversion. Red line is smoothed model, dividing the crust into 4-5 homogenous layers only, obtained by bifold forward modeling of both RFs and SWD. Error bounds on the Moho depth are shown by red dashed line. Black arrow represents depth of the Moho from forward modeling. ∆ is range of epicentral distance, ‘*b*’ is back-azimuthal range of stacked events and ‘*la*’, ‘*lo*’ are coordinates of Moho piercing point - point where the converted P to S phase pierces the Moho, for the average stacked RF. **(BOTTOM)** shows 2 km bin RFs along SW-NE profile L1-L1`passing NW of the Kashmir basin with distances reckoned from the MBT. Green line represents axis of the valley. Dashed lines at ~2 sec and ~7 sec represent dip in decollement and Moho NE of station BDP. RFs from regions which aren't discussed in the text are shaded by gray polygons. Figure was created using GMT (Generic mapping tool) software version 6.0.0 (http://gmt.soest.hawaii.edu/projects/gmt/wiki/Download) (Wessel, P. et al. 2019)


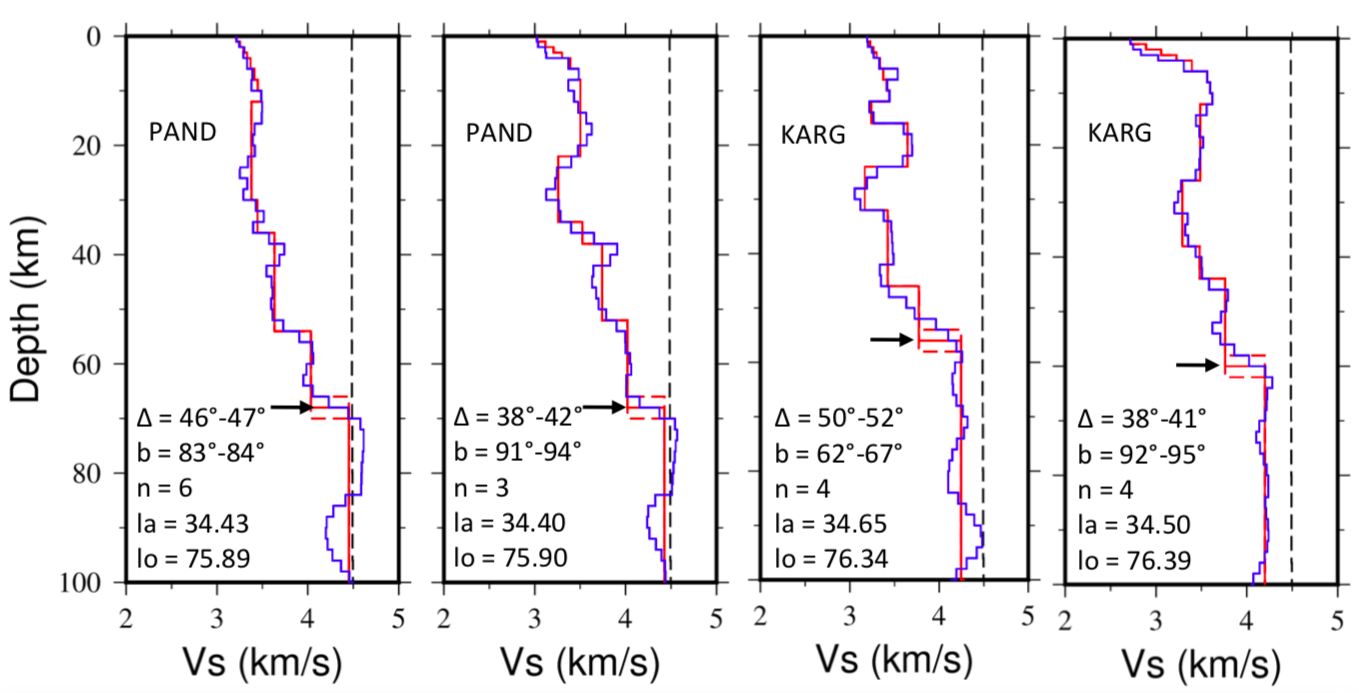

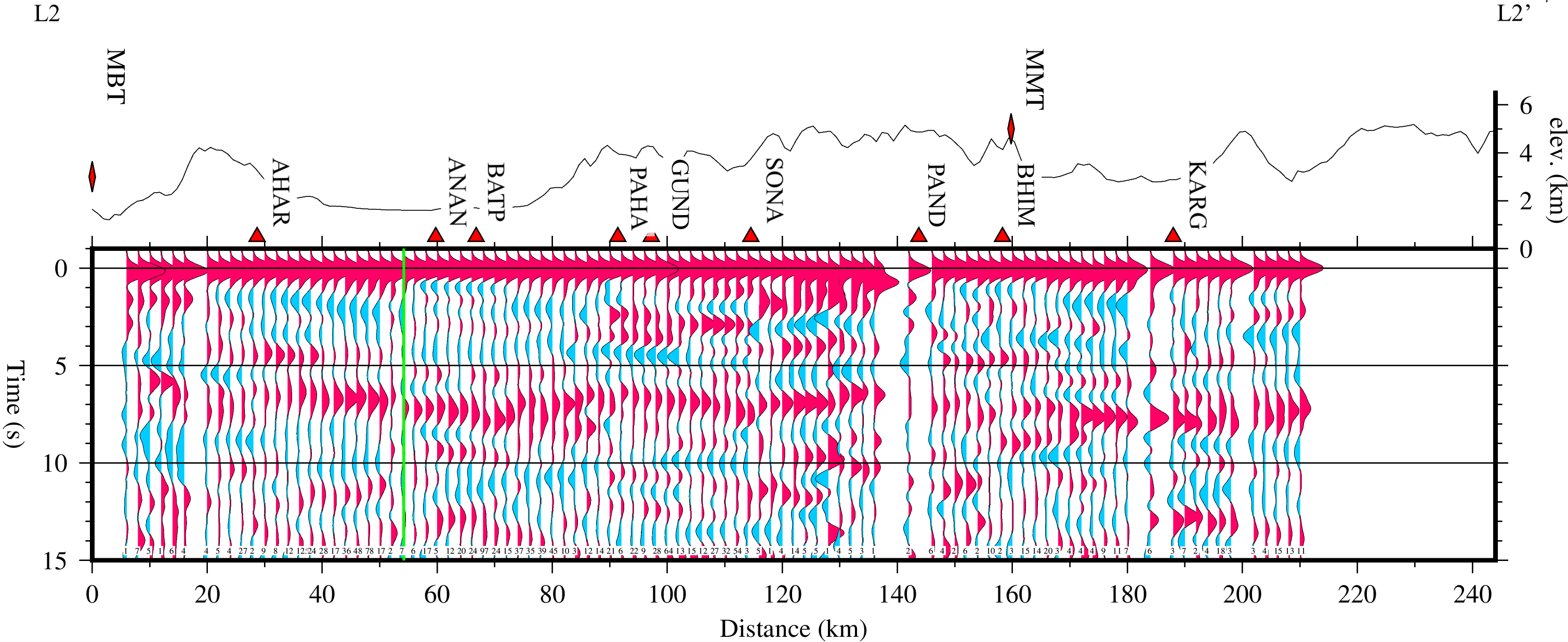


**Figure S4: (TOP)** Same as Figure S1 for stations PAND and KARG. The Moho piercing points for RFs at PAND, whose inversion is shown, lie close to location of BHIM. The Moho depths estimated here are ~66±2 km. However, ~15 km E/NE of station KARG the Moho upwarps to depth of ~60 km. **(BOTTOM)** shows cross-section along SW-NE arc-normal profile L2-L2`. Dashed line shows the upwarp in Moho as demonstrated by above 4 forward models. RFs from regions which aren't discussed in the text are shaded by a gray polygon. Figure was created using GMT (Generic mapping tool) software version 6.0.0 (http://gmt.soest.hawaii.edu/projects/gmt/wiki/Download) (Wessel, P. et al. 2019)

**
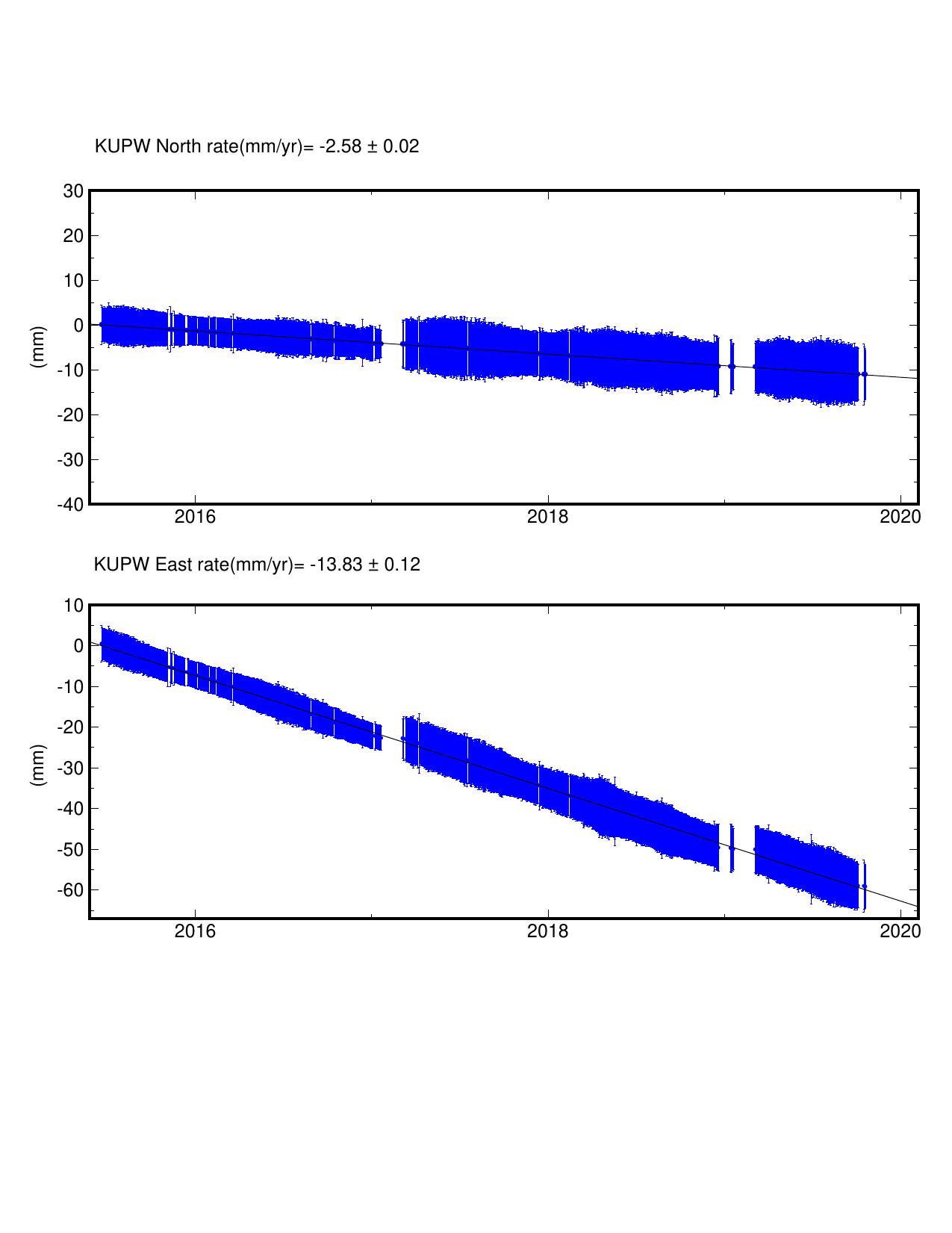
**

**Figure S5:** India fixed north and east coordinate time series of Kupwara (KUPW) cGPS site to extreme northwest of Kashmir valley for 4.5 years. Seasonal effects are removed from the coordinate time series. Figure was created using GMT (Generic mapping tool) software version 5.2.1 (ftp://ftp.soest.hawaii.edu/gmt/legacy/) (Wessel, P. et al. 2013)

**
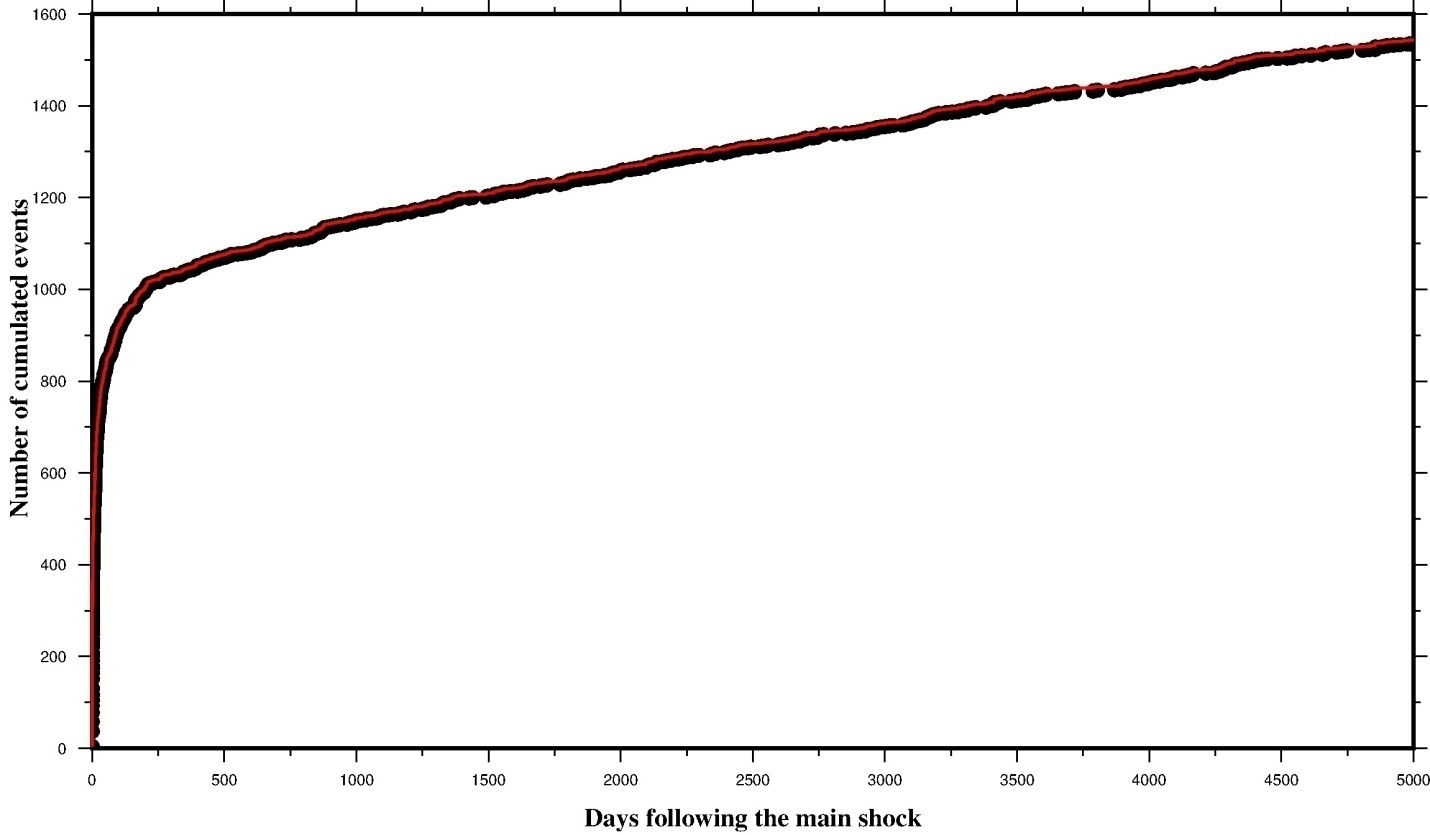
**

**Figure S6:** Black circles are cumulative number of events of M ≥ 3 within 100 km radius of the (source: <http://www.isc.ac.uk/iscbulletin/search/catalogue/>) October 2005 Muzaffarabad rupture for ~13.7 years after the earthquake covering the GPS measurements at Kupwara site (2015-2019). Trend line is denoted in Red. Figure was created using GMT (Generic mapping tool) software version 5.2.1 (ftp://ftp.soest.hawaii.edu/gmt/legacy/) (Wessel, P. et al. 2013)

**References:**

1. Mir, et al. Crustal structure beneath the Kashmir basin adjoining the Western Himalayan syntaxis, *Bull. Seismol. Soc.Am*., **107(5)**, 2443-2458 (2017).
2. Mir, Ramees R. Crustal Structure and Quantified Earthquake Hazard in the North WesternHimalaya, PhD thesis, pp 185, submitted to *Academy of Scientific and Innovative Research*, New Delhi (2020).
3. Kaila, K. L., Tripathi, K. M., Dixit, M. M. Crustal structure along Wular Lake-Gulmarg-Naosheara Profile Across PirPanjal Range of the Himalayas From Deep Seismic Soundings. *Geological Societyof India*, 706-719, nov. 1984. ISSN 0974-6889. <http://www.geosocindia.org/index.php/jgsi/article/view/65377> (1984).
4. Bollinger, L., Avouac, J.P., Cattin, R., Pandey,M.R. Stress buildup in the Himalaya. *J. Geophys. Res.***109**, B11405. http://dx.doi.org/10.1029/2003JB002911 (2004).
5. Cattin, R. and J. P. Avouac. Modeling mountain building and the seismic cycle in the Himalaya of Nepal, *J. Geophys. Res*. **105**, 13,389–13,407, doi: 10.1029/2000JB900032 (2000).
6. Wessel, P. et al. The Generic Mapping Tools version 6. *Geochemistry, Geophysics, Geosystems,* 20, 5556-5564. https://doi.org/10.1029/2019GC008515 (2019).
7. Paul, et al. Signatures of the Existence of Frontal and lateral ramp structures near the Kishtwar Window of the Jammu and Kashmir Himalaya: Evidence from Microseismicity and source mechanisms. *Geochemistry, Geophysics, Geosystems.***19,** 3097-3114 (2018).
8. Wessel, P., W. H. F. Smith., R. Scharroo., J. Luis and F. Wobbe. Generic Mapping Tools: Improved Version Released. *Eos. Trans. Am. Geophys. Union*. **94(45),** 409-410 doi: 10.1002/2013EO450001 (2013).
